# Altered Functional Topological Organization in Type-2 Diabetes Mellitus With and Without Microvascular Complications

**DOI:** 10.3389/fnins.2021.726350

**Published:** 2021-09-22

**Authors:** Dongsheng Zhang, Yang Huang, Jie Gao, Yumeng Lei, Kai Ai, Min Tang, Xuejiao Yan, Xiaoyan Lei, Zhen Yang, Zhirong Shao, Xiaoling Zhang

**Affiliations:** ^1^Department of MRI, Shaanxi Provincial People’s Hospital, Xi’an, China; ^2^Department of Graduate, Xi’an Medical University, Xi’an, China; ^3^Department of Clinical Science, Philips Healthcare, Xi’an, China

**Keywords:** type 2 diabetes mellitus, resting-state functional magnetic resonance imaging, graph theory, small-world, topological properties, microvascular complications

## Abstract

Microvascular complications can accelerate cognitive impairment in patients with type 2 diabetes mellitus (T2DM) and have a high impact on their quality of life; however, the underlying mechanism is still unclear. The complex network in the human brain is the physiological basis for information processing and cognitive expression. Therefore, this study explored the relationship between the functional network topological properties and cognitive function in T2DM patients with and without microvascular complications (T2DM-C and T2DM-NC, respectively). Sixty-seven T2DM patients and 41 healthy controls (HCs) underwent resting-state functional MRI and neuropsychological assessment. Then, graph theoretical network analysis was performed to explore the global and nodal topological alterations in the functional whole brain networks of T2DM patients. Correlation analyses were performed to investigate the relationship between the altered topological parameters and cognitive/clinical variables. The T2DM-C group exhibited significantly higher local efficiency (Eloc), normalized cluster coefficient (γ), and small-world characteristics (σ) than the HCs. Patients with T2DM at different clinical stages (T2DM-C and T2DM-NC) showed varying degrees of abnormalities in node properties. In addition, compared with T2DM-NC patients, T2DM-C patients showed nodal properties disorders in the occipital visual network, cerebellum and middle temporal gyrus. The Eloc metrics were positively correlated with HbA1c level (*P* = 0.001, *r* = 0.515) and the NE values in the right paracentral lobule were negatively related with serum creatinine values (*P* = 0.001, *r* = −0.517) in T2DM-C patients. This study found that T2DM-C patients displayed more extensive changes at different network topology scales. The visual network and cerebellar may be the central vulnerable regions of T2DM-C patients.

## Introduction

Type 2 diabetes mellitus (T2DM) is characterized by insulin resistance and chronic hyperglycemia, which has become the most common metabolic disease worldwide (Chatterjee et al., 2017; Ogurtsova et al., 2017). Long-term hyperglycemia causes renal mesangial cell expansion and accelerates endothelial cell apoptosis through multiple pathways, and ultimately leads to various microvascular complications including diabetic nephropathy, retinopathy, and peripheral neuropathy (Satirapoj, 2012; Singh et al., 2020). Although T2DM often leads to a variety of cognitive dysfunctions (Macpherson et al., 2017; Karvani et al., 2019), multiple clinical studies have found that microvascular complications can accelerate cognitive impairment and have a high impact on patients’ quality of life and overall life expectancy (Exalto et al., 2014; Li et al., 2018; Faselis et al., 2020). Therefore, it is very important to explore the neural mechanism of microvascular complications affecting the cognitive impairment of T2DM patients.

Previous neuroimaging studies used different methods to explore the relationship between abnormal brain function and cognitive function in T2DM patients with different microvascular complications. Multiple studies (Wang et al., 2017, 2019; Liao et al., 2019) have confirmed abnormal neuronal activity in the bilateral occipital lobes, cerebellum, and other brain regions in T2DM patients with diabetic nephropathy and retinopathy. These studies suggest that the damage to the occipital cortex may be related to defects in the patient’s visual function. In addition, decreased mean voxel-mirrored homologous connection values in the bilateral middle occipital gyrus were positively correlated with the urinary protein-to-creatinine ratio in patients with diabetic nephropathy and retinopathy (Wang Y. et al., 2020). Huang et al. (2019) found that patients with diabetic retinopathy (DR) showed abnormal nodal centralities, and functional disconnections were mainly located in the default-mode network, visual network, and sensorimotor network. Other studies have shown that patients with diabetic peripheral neuropathy have abnormal neuron activity in the somatosensory and cognitive brain regions (Zhang et al., 2019); bilateral pre and postcentral gyrus functional connectivity disorders (Cauda et al., 2009). It has been speculated that the central nervous system may contribute to painful diabetic neuropathy. Although the above studies have shown that the damaged occipital lobe visual cortex and the sensorimotor cortex are related to the microvascular complications of T2DM, dysfunctions in the cerebellum and other brain regions have also been noted. However, almost all of the above-mentioned studies only focused on the abnormal changes of brain function of T2DM patients with microvascular complications (T2DM-C). It is not clear how characteristic changes exist in the above-mentioned brain regions and global brain functions in T2DM patients in different clinical stages (with and without microvascular complications). Although previous studies (Fang et al., 2018; Zhuo et al., 2019) have explored the changes in gray matter volume and white matter microstructure in T2DM patients with and without microvascular complications (T2DM-C and T2DM-NC, respectively), it is suggested that damage to the brain structure may progress gradually with the emergence of microvascular complications. However, T2DM-related changes in brain function may be a complex process from compensation to impairment (decompensation) (van Bussel et al., 2016; Zhang et al., 2021). Therefore, a comprehensive understanding of the abnormal changes of brain function in patients at different clinical stages will help us identify new methods to evaluate and treat cognitive impairment in T2DM.

Altered neuronal connectivity might be a crucial step in the putative pathway of microvascular dysfunction leading to cognitive decline (Filippi et al., 2013). Therefore, choosing a method that can comprehensively reflect the changes in brain function at different scales of the whole brain may be more helpful to reveal the characteristics of brain damage in T2DM-C patients. There is growing evidence to show that the complex network in the human brain forms the physiological basis for all information processing and cognitive expression (Reijneveld et al., 2007; Liao et al., 2017). The resting-state functional magnetic resonance imaging (rs-fMRI) analysis method based on graph theory can reflect the interaction and integration of brain neuron clusters or brain regions at different levels and scales (Stam and Reijneveld, 2007; Bullmore and Sporns, 2009). This method can fully and effectively reflect the changes in the topology of the human brain network feature (Minati et al., 2013). In recent years, this method has been widely used to explore the pathogenesis of many neuropsychiatric diseases such as AD, epilepsy, and schizophrenia (Alexander-Bloch et al., 2013; Reijmer et al., 2013; Fallahi et al., 2020).

Therefore, in the present study, we used rs-fMRI with graph theoretical analysis to explore the characteristic changes of functional brain network topological properties in T2DM-C and T2DM-NC patients. We speculate that there are different degrees of abnormal brain network topology properties in different clinical stages (with and without microvascular disease) in T2DM patients. T2DM-C patients have more significant changes in diversity network topology properties, especially in the visual network and sensorimotor network, and the changed brain network topology properties may be related to clinical and cognitive variables.

## Materials and Methods

### Participants

Sixty-nine subjects (39 T2DM-C and 30 T2DM-NC) were recruited from the Department of Endocrinology of Shaanxi Provincial People’s Hospital in this study. Forty-one healthy controls (HCs) matching the demographic data of T2DM patients were employed from the health examination center of our hospital. All subjects were between 45 and 70 years of age, right-handed, and had at least 6 years of education. The diagnostic criteria of T2DM patients were based on the 2014 American Diabetes Association guidelines. Patients with T2DM were on stable therapy (diet, oral medications, and/or insulin). DR was defined as present if any of the following lesions were detected: retinal microaneurysms, hemorrhages, hard exudates, soft exudates, neovascularization, or evidence (also history) of laser photocoagulation (Fang et al., 2018). Diabetic nephropathy was diagnosed using the following criteria (Stevens et al., 2013): (1) urine albumin/creatinine ratio (ACR) > 300 mg/g or ACR range: 30–300 mg/g, and (2) pathological examination of the kidney suggestive of diabetic nephropathy. The patients meeting any two of the following criteria were regarded as having diabetic peripheral neuropathy (Tesfaye et al., 2010): (1) presence of typical neuropathic sensory symptoms; (2) a symmetric decrease in distal sensation or loss of ankle reflexes; and (3) abnormal results of nerve conduction studies. At least one microvascular complication was assigned to T2DM-C patients. Patients without any kind of microvascular complications were assigned to the T2DM-NC group. Patients were excluded from the study if they had a history of hypoglycemia (blood glucose concentration of <3.9 mmol/L). The exclusion criteria in all subjects were as follows: (1) severe claustrophobia or contraindications to MRI; (2) alcoholism, Parkinson’s disease, major depression, brain injury, epilepsy, or other neurological or psychiatric disorders; (3) presence of another primary, secondary, or congenital kidney disease (e.g., acute nephritis, chronic nephritis, lupus nephritis, gouty kidney, traumatic renal injury, IgA nephropathy, and renal artery disease); (4) presence of another ocular diseases; and (5) a Mini-Mental State Examination (MMSE) score < 24.

Every subject arrived at the department for MRI at 6:30–7:00 pm after dinner and controlled their blood glucose according to their doctor’s orders on the day of the scan. MRI was performed after approximately 30 min of structured clinical interview and a series of psychological tests. Only one patient was scanned each day to ensure that everyone completed the examination with a relatively stable blood glucose. The test procedure and scan time of HCs was the same as those of T2DM patients. All subjects were awake during the MRI and did not experience any discomfort. The study was approved by the ethics committee of Shaanxi Provincial People’s Hospital. The study protocol was explained to all subjects, and all subjects provided written informed consent before participation.

### Clinical Data and Neuropsychological Tests

We obtained the medical history and clinical data of the patients. The clinical data of the HCs were collected from the outpatient medical examination center and included weight, height, blood pressure, and body mass index (BMI). Blood pressure was measured at three different times during the day and then averaged. Glycated hemoglobin (HbA1c), fasting blood glucose (FBG), triglyceride, cholesterol, low-density lipoprotein cholesterol (LDL-C), serum urea, serum creatinine, urine microalbumin, urinary creatinine, and ACR were measured by standard laboratory testing assays. The MMSE and Montreal Cognitive Assessment (MoCA) scores were used to assess general cognitive function. Information processing speed and attention were tested by the Trail Making Test A (TMT-A). Executive function and visuospatial skills were evaluated by the clock-drawing test (CDT). The neuropsychological tests were performed by a psychiatrist with > 5 years’ work experience.

### Data Acquisition and Preprocessing

All participants underwent MRI scanning on a 3.0T MR scanner (Philips Ingenia, Best, Netherlands) with a 16-channel-phased array head coil in the Department of MRI of Shaanxi Provincial People’s Hospital. Conventional T2-weighted imaging (T2WI) and fluid attenuated inversion recovery (FLAIR) scans were used to exclude visible brain lesions. Rs-fMRI images were obtained using a gradient-echo planar sequence with the following parameters: repetition time (TR) = 2,000 ms, echo time (TE) = 30 ms, slices = 34, thickness = 4 mm (no gap), flip angle (FA) = 90°, field of view (FOV) = 230 mm × 230 mm, and matrix = 128 × 128; further, 200 volumes were acquired in each scan. Sagittal 3-dimensional T1-weighted images (T1WI) were obtained using a fast spoiled gradient echo sequence with the following parameters: TR = 7.5 ms, TE = 3.5 ms, FA = 8°, FOV = 250 mm × 250 mm, matrix = 256 × 256, slice thickness = 1 mm (no gap), and 328 sagittal slices. All participants were instructed to close their eyes but stay awake throughout the scan.

The rs-fMRI data were preprocessed using the DPABI^1^ software package (Yan et al., 2016). The first 10 time points were discarded to ensure stabilization of the magnetic field. The slice-timing and realignment of head motion correction were performed for the remaining 190 time points. Two participants were excluded from this study whose head motion was > 1.5 mm or translation was > 1.5° rotation in the T2DM-C group. Then, the images were spatially normalized to the standard MNI space using the EPI template (resampling voxel size = 3 mm × 3 mm × 3 mm) and smoothed using a Gaussian kernel with a 6-mm full width at half maximum (FWHM). Moreover, “scrubbing” method was used to reduce the effect of high head motion (Power et al., 2012). We calculated the frame-wise displacement (FD) and set an FD threshold for bad volumes as 0.2 mm. The bad volumes were scrubbed which the FD values were greater than 0.2 mm, as well as one forward volume and two back volumes of the bad volumes. Then, each bad volume was modeled as a regressor in the model regression (Yan et al., 2013). Additionally, we regressed out 24 head motion parameters, the linear trend signal, cerebrospinal fluid signals, and white matter signals. Finally, a temporal-band filter (0.01–0.08 Hz) was used to regress the effect of physiological noise.

### Network Construction and Analysis

The whole-brain regions of each participant were parcellated into 116 regions, containing 90 brain regions and 26 cerebellar regions, according to the Automated Anatomically Labeling (AAL) atlas by toolbox GRETNA^2^ (Wang et al., 2015). Subsequently, we extracted the mean time series of each region and calculated the Pearson correlation coefficients between every pair of regional time series. Fisher’s Z transformation was used to convert correlation coefficients to Z-values for improving normal distribution; thus, a 116 × 116 Z-value correlation matrix was generated for each participant. As there was no golden standard for selecting a single threshold, and sparsity (Sp) was defined as the ratio of the number of existing edges divided by the maximum possible number of edges in a network. The sparsity ensured the same number of edges for each network and minimized the effects of possible discrepancies in the overall correlation strength between the group (He et al., 2009a; Wang J. et al., 2020). Therefore, we applied a common wide range of sparsity threshold (Chen et al., 2018) (0.05 < S < 0.5, step = 0.01) to convert the Z-matrix into a binary connection matrix graph for further analyses, and a graphic model of the brain functional network was constructed.

In this study, we defined each brain region as a node and each functional connectivity between two nodes as an edge to construct a binary brain network using the GRETNA toolbox (Wang et al., 2015). Then, we calculated several indices in graph theory to characterize the topological properties of brain functional network, which can estimate the functional segregation and integration of the brain network, including the clustering coefficient (Cp) and characteristic path length (Lp). The network indices Cp and Lp were normalized by comparable values of 1,000 random networks to obtain the normalized clustering coefficient (γ = Cp real/Cp rand > 1), normalized characteristic path length (λ = Lp real/Lp rand≈ 1), and the ratio of γ and λ was defined as the small-worldness (σ = γ/λ > 1). Additionally, we calculated the global efficiency (Eglob) and the local efficiency (Eloc) parameters to measure the network efficiency of transmitting information at the local and global levels. Furthermore, the nodal metrics of whole-brain network including degree centrality (DC), betweenness centrality (BC), and nodal efficiency (NE) were calculated in each individual node of the brain network. DC reflects the importance of the node or brain region in the whole brain network, while BC characterizes the ability of the node to influence the entire network. Moreover, NE indicates the efficiency of the parallel information transmission capability of the node in the network. Finally, we calculated the area under the curve (AUC) for each network metric over all sparsity range to detect the topological characterization of the brain functional connectome (He et al., 2009b; Liu et al., 2016).

### Statistical Analysis

The chi-square (χ^2^) test was used to analyze the sex-based differences among the three groups. One-way analysis of variance (ANOVA) was calculated in other demographic clinical and cognitive scores. An independent two-sample *t*-test was used to compare disease duration, serum urea, serum creatinine, urine microalbumin, urinary creatinine, and ACR between the T2DM-C and T2DM-NC patient groups. All the above analyses were performed using SPSS Statistics version 24 (IBM Corporation, Armonk, NY, United States), and the significance threshold was set at *P* < 0.05.

For comparisons of the global network topological metrics in AUCs (Cp, Lp, γ, λ, σ, Eg, and Eloc) and nodal topological metrics (DC, BC, and NE) among the three groups, ANOVA (FDR correction, *P* < 0.05) was applied with age, gender, and FD values as covariates. *Post hoc* (FDR correction, *P* < 0.05) analysis was performed in pairs within the areas identified by ANOVA to compare the differences between each group with age, gender, and FD values as covariates. Moreover, to explore the relationships between topological properties of the brain network and clinical/cognitive scores, partial correlation between topological properties and cognitive/clinical variables was further performed in patient groups, removing age, gender, and FD values as covariates. We used a statistical significance level of *P* < 0.05, and the FDR was used to correct for multiple comparisons.

## Results

### Clinical Data and Neuropsychological Tests

Two T2DM-C patients were excluded for excessive motion; thus, 37 T2DM-C, 30 T2DM-NC, and 41 HCs were included in the final analyses. The demographic, clinical, and neuropsychological data of the participants are presented in Table 1. There were no significant differences among the three groups in age, sex, years of education, BMI, triglyceride, cholesterol, blood pressure, MMSE score and mean FD values (*P* > 0.05), and we also observed no statistical differences in the serum urea, serum creatinine, and urinary creatinine concentration between the two groups of diabetic patients (*P* > 0.05). As expected, patients with T2DM had higher levels of HbA1c and FBG than the HCs (all *P* < 0.001). In terms of cognitive performance, both T2DM-C and T2DM-NC patients had poorer MoCA scores than the HCs (all *P* < 0.001); T2DM-C patients had poorer TMT-A scores than the HCs (*P* < 0.05). In addition, T2DM-C patients had poorer CDT scores than the T2DM-NC patients (*P* < 0.05). Furthermore, the T2DM-C patients showed higher urine microalbumin and ACR concentration than the T2DM-NC patients (*P* < 0.05).

**TABLE 1:** Demographic, clinical, and neuropsychological features of the subjects.

Variables	T2DM-C (*n* = 37)	T2DM-NC (*n* = 30)	HC (*n* = 41)	T/F/χ2 value	*P*-value
Male/female	19/18	17/13	22/19	0.593	0.441^#^
Age (years)	53.76 ± 1.14	55.00 ± 0.94	53.93 ± 0.80	0.767	0.467
Educational level (years)	13.11 ± 0.47	13.63 ± 0.48	14.27 ± 0.54	1.491	0.250
Diabetes duration (months)	91.18 ± 11.36	72.50 ± 54.93	–	1.972	0.233^&^
Systolic BP (mmHg)	122.59 ± 2.58	120.67 ± 3.15	120.48 ± 3.93	0.876	0.419
Diastolic BP (mmHg)	81.03 ± 2.07	80.37 ± 2.31	79.44 ± 2.87	0.940	0.394
BMI (kg/m^2^)	24.91 ± 0.45	24.57 ± 0.50	24.07 ± 0.40	0.967	0.384
FBG (mmol/L)	9.32 ± 0.53^a^	7.61 ± 0.48^a^	5.14 ± 0.39	40.792	< 0.001*
HbA1c (%)	8.80 ± 0.41^a^	7.84 ± 0.40^a^	5.58 ± 0.27	24.012	< 0.001*
Triglyceride (mmol/L)	1.67 ± 0.26	1.56 ± 0.16	1.47 ± 0.14	1.703	0.187
Total cholesterol (mmol/L)	4.42 ± 0.16	4.32 ± 0.29	4.67 ± 0.21	2.049	0.134
LDL (mmol/L)	2.32 ± 0.11	2.34 ± 0.19	2.53 ± 0.15	2.305	0.105
Serum urea (mmol/L)	4.51 ± 0.25	4.93 ± 0.26	–	–1.140	0.259^&^
Serum creatinine (μmol/l)	59.39 ± 0.11	55.56 ± 0.19	–	0.096	0.368^&^
Urine microalbumin (mg/L)	99.17 ± 31.43	5.68 ± 0.97	–	2.382	0.018^&^*
Urinary creatinine (mg/L)	1002.7 ± 111.4	939.1 ± 101.7	–	–1.250	0.491^&^
ACR (mg/g)	104.67 ± 29.30	6.57 ± 1.19	–	2.744	0.008^&^*
MMSE	28.08 ± 0.34	27.83 ± 0.35	28.60 ± 0.27	1.537	0.220
MoCA	25.23 ± 0.54^a^	25.00 ± 0.45^a^	26.78 ± 0.31	4.978	0.010*
CDT	19.73 ± 1.39^b^	23.87 ± 1.45	22.61 ± 1.03	2.664	0.070
TMT-A	83.80 ± 5.73^a^	79.56 ± 5.29	66.50 ± 4.42	3.529	0.033*
FD	0.08 ± 0.01	0.08 ± 0.01	0.07 ± 0.01	0.126	0.882

Data are presented as mean ± standard deviation or number (%) unless otherwise indicated. BMI, body mass index; FBG, fasting blood glucose; HbA1c, glycated hemoglobin; LDL-C, low-density lipoprotein cholesterol; ACR, urine albumin/creatinine ratio; MMSE, Mini-Mental State Examination; MoCA, Montreal Cognitive Assessment; CDT, clock-drawing test; TMT-A, Trail-Making Test A; FD, framewise displacement. ^#^*P* for the χ^2^ test; ^&^*P* for the two-sample *t*-test; *P < 0.05. ^*a*^*Post hoc* paired comparisons show significant differences compared with HCs. ^*b*^*Post hoc* paired comparisons show significant differences compared with T2DM-NC patients.

### Global Topological Properties

**FIGURE 1:**
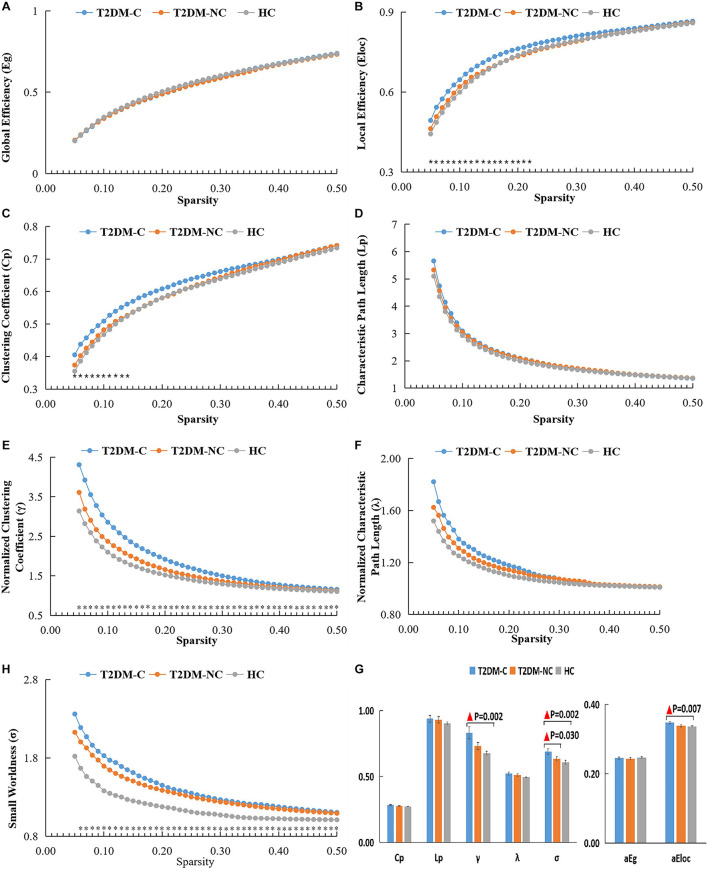
The global topological metrics of the whole-brain functional network of the three groups. Graphs **(A–G)** show the small-word properties and network efficiency under different sparsity (0.05 < S < 0.5). The AUC of small-world properties and network efficiency over full sparsity range is shown by histogram graphs **(H)**. The symbol “▲” denotes statistical significance. Cp, clustering coefficient; Lp, characteristic path length; γ, normalized clustering coefficient; λ, normalized shortest path length; Eg, global efficiency; Eloc, local efficiency; AUC, area under curve.

The global topological metrics including clustering coefficient (Cp), characteristic path length (Lp), normalized clustering coefficient (γ), normalized characteristic path length (λ), small-worldness (σ), global efficiency (Eg), and local efficiency (Eloc) in each sparsity threshold range of the three groups are presented in Figures 1A–G. Over the defined sparsity range (0.05 < sparsity < 0.5, step = 0.01), the ANOVA and *post hoc* analyses were performed to detect the differences in the AUC of global network properties among the three groups. The detailed description of the global topological properties (Cp, Lp, γ, λ, σ, Eg, Eloc) over full sparsity range (0.05–0.5) are shown in Figure 1H. In summary, small-worldness of whole-brain functional networks were observed in the three groups, and there was no statistical difference in Cp, Lp, λ and Eg among three groups. However, the T2DM-C group exhibited significantly higher Eloc, normalized cluster coefficient (γ), and small-world characteristics (σ) than the HCs. Compared with T2DM-NC patients, T2DM-C patients exhibited significantly higher small-world characteristics (σ). There was no statistical difference in all global topological metrics between the T2DM-NC patients and the HCs.

### Nodal Topological Properties

**FIGURE 2:**
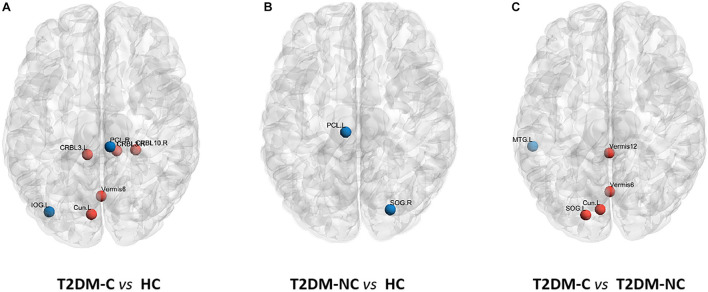
Distribution of nodes properties with statistical differences between groups. **(A)** The distribution of the significant nodal properties between T2DM-C and HC group. **(B)** The distribution of the significant nodal properties between T2DM-NC and HC group. **(C)** The distribution of the significant nodal properties between T2DM-C and T2DM-NC group. Red color indicates increased nodal properties, and the blue color indicates decreased nodal properties.

The significant differences in nodal topological metrics of AUC over all sparsity thresholding among the three groups are shown in Table 2. Specifically, compared with the HCs, the T2DM-C patients showed increased BC values in the left cuneus and vermis 6. Compared with the T2DM-NC group, T2DM-C patients showed increased BC values in the left cuneus and left superior occipital gyrus (SOG). Moreover, compared with the HCs, the T2DM-C patients showed decreased DC values in the right paracentral lobule (PCL) and left inferior occipital gyrus and increased DC values in the bilateral cerebellum 3, right cerebellum 10, vermis 6, and T2DM-NC patients showed decreased DC values in the right SOG and the left PCL. Compared with the T2DM-NC group, T2DM-C patients showed decreased DC values in the left middle temporal gyrus. Additionally, compared with HCs, T2DM-C patients showed decreased NE values in the right PCL and left inferior occipital gyrus and increased NE values in the bilateral cerebellum 3. T2DM-NC patients had decreased NE values in the right SOG. Compared with the T2DM-NC group, T2DM-C patients showed incresaed NE values in the vermis 1, 2. The distribution of node properties with statistical differences between groups were represented in the Figure 2.

**TABLE 2:** The significant differences in nodal topological metrics of AUC.

Nodal metrics	Regions (AAL)	ANOVA	T2DM-C VS. HC	T2DM-NC VS. HC	T2DM-C VS. T2DM-NC
BC	Cuneus_L	*P* = 0.001	*P* = 0.010 *T* = 3.33		*P* = 0.008 *T* = 3.19
	Occipital_Sup_L	*P* = 0.001			*P* = 0.008 *T* = 3.21
	Vermis_6	*P* = 0.006	*P* = 0.011 *T* = 3.08		
DC	Occipital_Sup_R	*P* = 0.008		*P* = 0.024 *T* = −3.02	
	Occipital_Inf_L	*P* = 0.016	*P* = 0.007 *T* = −3.05		
	Paracentral_Lobule_L	*P* = 0.008		*P* = 0.024 *T* = −3.04	
	Paracentral_Lobule_R	*P* = 0.006	*P* = 0.007 *T* = −3.05		
	Temporal_Mid_L	*P* = 0.001			*P* = 0.003 *T* = −3.90
	Cerebelum_3_L	*P* = 0.008	*P* = 0.007 *T* = 3.07		
	Cerebelum_3_R	*P* = 0.003	*P* = 0.007 *T* = 3.26		
	Cerebelum_10_R	*P* = 0.001	*P* = 0.007 *T* = 3.32		
	Vermis_6	*P* = 0.003	*P* = 0.007 *T* = 3.38		*P* = 0.015 *T* = 3.20
NE	Occipital_Sup_R	*P* = 0.007		*P* = 0.010 *T* = −3.44	
	Occipital_Inf_L	*P* = 0.010	*P* = 0.007 *T* = −3.19		
	Paracentral_Lobule_R	*P* = 0.005	*P* = 0.007 *T* = −3.17		
	Cerebelum_3_L	*P* = 0.006	*P* = 0.007 *T* = 3.14		
	Cerebelum_3_R	*P* = 0.002	*P* = 0.006 *T* = 3.46		
	Vermis_1_2	*P* = 0.009			*P* = 0.009 *T* = 3.01

*BC, betweenness centrality; DC, degree centrality; NE, nodal efficiency; L, left; R, right.*

### Correlation Analysis

**FIGURE 3:**
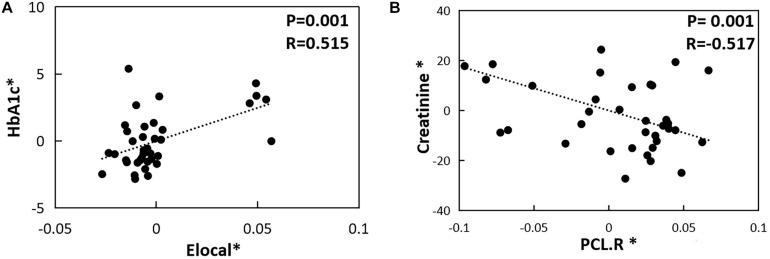
The correlations between topological properties and cognitive/clinical variables. **(A)** Correlation between Eloc metrics and HbA1c level in the T2DM-C group (*P* = 0.001, *r* = 0.515). **(B)** Correlation between NE values in the right PCL and serum creatinine values in the T2DM-C group (*P* = 0.001, *r* = –0.517) The asterisk (*) indicated coordinate values controlling for the influence of gender, age, and FD.

In the T2DM-C group, the Eloc metrics were positively correlated with HbA1c level (*P* = 0.001, *r* = 0.515) (Figure 3A). Furthermore, the NE values in the right PCL were negatively related with serum creatinine values (*P* = 0.001, *r* = −0.517) (Figure 3B). There was no correlation between the network topology properties with clinical and neuropsychological variables in the T2DM-NC group and all T2DM patients.

## Discussion

In this study, the rs-fMRI method based on graph theory was used to explore the changes in the topological properties of the brain function network in T2DM-C and T2DM-NC patients. Compared with HCs, the T2DM-C groups had significantly increased small-world network parameters, and the disorder of the nodal topology was more obvious. The results suggested that T2DM patients with different clinical stages had characteristic brain network topology changes. Patients with T2DM-C have more extensive brain network topology reorganization.

### Altered Small-World Networks in the T2DM-C Patients

The topological organization of the normal human brain has small-world properties (Reijneveld et al., 2007). Small-world property refers to an optimal balance between local specialization and global integration, which enables efficient segregated and integrated information processing in the whole-brain network (Watts and Strogatz, 1998). In the current study, T2DM-C patients displayed higher normalized cluster coefficient (γ), small-world characteristics (σ), and Eloc properties than the HCs, and T2DM-NC patients were intermediate between those two groups. This may suggest that the connections between nodes and adjacent brain regions have increased, and the local network efficiency more optimized in patients with T2DM, which is consistent with previous T2DM-related findings (Qin et al., 2019; Xu et al., 2019). However, it is interesting that the Eg of T2DM patients did not increase. This may indicate that in order to maintain the overall network efficiency at a relatively normal level, the local network efficiency shows a compensatory increase. In this study, all T2DM patients had an MMSE score > 24. The previous study (Filippi et al., 2017; Wang D. et al., 2020) found that Eloc increased in patients with mild cognitive impairment (MCI) but decreased in those with dementia. Therefore, we speculate that before dementia in T2DM patients, Eloc may show a compensatory increase to maintain cognitive function. Moreover, this compensatory phenomenon is more significant in T2DM-C patients. Furthermore, a study (Liu et al., 2018) found that T2DM patients without dementia have more short-range connections and less long-range connection density. The brain regions with short-range functional connectivity density are usually dedicated to modular information processing and are similar to Eloc in small-world networks (Sepulcre et al., 2010), which further confirms our speculation. The positive correlation between Eloc and HbA1c may indicate that persistent hyperglycemia is responsible for a compensatory increase in Eloc.

### Altered Nodal Topological Properties in T2DM Patients at Different Clinical Stages

The occipital lobe visual network is one of the most vulnerable brain regions in patients with T2DM (Macpherson et al., 2017). The cuneus participates in the visual space process and has the function of visual orientation, while SOG has the function of visual perception (Coppen et al., 2018). Our research found that T2DM-C patients had higher BC values in the left cuneus and SOG than the T2DM-NC patients. This indicates that more information flows through those nodes in the visual network, which may be due to the extensive reorganization or interruption in the visual network of T2DM-C patients. Previous studies (Wang et al., 2017; Huang et al., 2019) have found that the ALFF value and the node properties of the right cuneus increased in DR patients. This study also found that multiple node properties in the visual network of T2DM patients at different clinical stages undergo abnormal changes, but T2DM-C patients have more disordered nodes, which further confirms that the visual network may be a vulnerable region in T2DM-C patients. Studies have suggested that abnormal neural activities in the visual network may affect spatial memory (Cui et al., 2014) and general cognitive functions (Wang et al., 2017). This may be one of the reasons for the lower CDT and MoCA scores in T2DM-C patients. In addition, the node properties of the right SOG decreased in T2DM-NC patients compared with HCs, which may indicate that the visual network is dysfunctional before the patient shows obvious complications. Previous studies have shown that the volume of gray matter in the occipital lobe is reduced in T2DM patients without retinopathy (Ferreira et al., 2017), which supports our speculation to a certain extent.

The PCL belongs to the sensorimotor cortex. This study found that the node properties of the sensorimotor cortex were decreased in T2DM patients at different clinical stages. Previous studies (Zhang et al., 2019; Roy et al., 2020) confirmed that the sensorimotor cortex (precentral gyrus, postcentral gyrus, and supplementary motor area) of patients with peripheral neuropathy had reduced gray matter volume and abnormal neuronal activity, suggesting that diabetic neuropathy is closely associated with abnormal central sensorimotor function. But this study found that the NE values in the right PCL were negatively related with serum creatinine values in patients with T2DM-C, it seems to suggest that the right PCL is also affected by renal function impairment. A study has found that patients with end-stage renal disease also have abnormalities in the sensorimotor cortex (Chen et al., 2020). Therefore, we speculate that the right PCL may be dually affected by diabetic peripheral neuropathy and nephropathy. However, it is interesting that the sensorimotor cortex of T2DM-NC patients still has decreased node properties, which may indicate that the central sensorimotor cortex has abnormal changes before the occurrence of microvascular complications.

Recent studies (Habas et al., 2009; Stoodley and Schmahmann, 2009) have confirmed that different cerebellar subregions are involved in multiple cognitive functions. Qin et al. (2019) found that multiple nodal topological properties of the cerebellum increased in patients with T2DM, and our results were partly consistent. Previous studies have found that the cerebellum has abnormal neuronal activity in DR patients (Wang et al., 2017; Liao et al., 2019), and the ALFF value of both anterior and posterior lobes of the cerebellum is increased in patients with diabetic nephropathy and retinopathy (Wang et al., 2019). In addition, decreased cerebellar gray matter volume in patients with peripheral neuropathy is correlated with walking speed and stride duration variability (Manor et al., 2012). The above studies show that the cerebellum is one of the vulnerable regions in any kind of microvascular disease. Our study found that T2DM-C patients have higher node properties in multiple subregions of the cerebellum than T2DM-NC patients and HCs, which suggests that the cerebellar network topology is disordered. This further confirms that the cerebellum may be a central damage target of T2DM-C patients. In addition, multiple studies (Ruet et al., 2014; Moroso et al., 2017) suggest that abnormal cerebellar structure and function are related to information processing speed impairment, which may be responsible for worse TMT-A score of patients with T2DM-C.

T2DM-C patients had lower DC and NE properties in the left middle temporal gyrus than T2DM-NC patients. However, compared with HCs, there were no significant differences in the node properties of the left middle temporal gyrus of T2DM patients at different clinical stages. This seems to be a contradictory result, but previous studies (Sun et al., 2017) have found that there is a U-shaped change in the left middle temporal gyrus and middle frontal gyrus in patients with and without dementia-related genotypes in MCI. It has been proven that even in the same disease, under different influencing factors, the changes in brain function are still complicated. Therefore, we speculate that the brain function changes of the left middle temporal gyrus in patients with T2DM at different clinical stages may be a complex non-linear process. Of course, this needs to be confirmed by further longitudinal studies with a larger sample size in future.

### Limitations

Our study has some limitations. First, this is a cross-sectional study with a small sample size, which may have a certain impact on the statistical power of the results. In the future, we plan to expand the sample size and use longitudinal studies to further explore the characteristics of brain function network changes in T2DM patients in different clinical stages. Second, the treatment plan for T2DM differed between patients. Different drugs may have had a certain bias on the study results, but this would be difficult to avoid. Third, there is no correlation between the network properties and cognitive scales in this study, which may be because of lack of comprehensiveness of our cognitive scales. However, previous studies (Berlot et al., 2016; Xue et al., 2020) have confirmed that disorder of network topology properties is closely related to cognitive dysfunctions.

## Conclusion

This study found that T2DM-C patients displayed more extensive changes at different network topology scales. Visual network and cerebellar cortices may be the central vulnerable regions of T2DM-C patients. This provides some imaging evidence for understanding the neural mechanisms of cognitive impairment in patients with T2DM at different clinical stages.

## Data Availability Statement

The raw data supporting the conclusions of this article will be made available by the authors, without undue reservation.

## Ethics Statement

The studies involving human participants were reviewed and approved by the Ethics Committee of Shaanxi Provincial People’s Hospital. The patients/participants provided their written informed consent to participate in this study.

## Author Contributions

DZ and YH drafted the manuscript and designed the experiment. YH performed the statistical analysis. JG and XL contributed to performing the experiments and revised the manuscript. YL, MT, XY, and ZS collected the data. KA and ZY provided technical support. XZ made contributions to the design of the experiment and revised the manuscript. All authors read and approved the final manuscript.

## Conflict of Interest

The authors declare that the research was conducted in the absence of any commercial or financial relationships that could be construed as a potential conflict of interest.

## Publisher’s Note

All claims expressed in this article are solely those of the authors and do not necessarily represent those of their affiliated organizations, or those of the publisher, the editors and the reviewers. Any product that may be evaluated in this article, or claim that may be made by its manufacturer, is not guaranteed or endorsed by the publisher.

## Footnotes

1
http://rfmri.org/dpabi/

2
http://www.nitrc.org/projects/gretna/
